# Nanoliposomes containing limonene and limonene-rich essential oils as novel larvicides against malaria and filariasis mosquito vectors

**DOI:** 10.1186/s12906-022-03624-y

**Published:** 2022-05-19

**Authors:** Alireza Sanei-Dehkordi, Mohammad Djaefar Moemenbellah-Fard, Mostafa Saffari, Elham Zarenezhad, Mahmoud Osanloo

**Affiliations:** 1 Department of Medical Entomology and Vector Control, School of Health, Hormozgan University of Medical Sciences, Bandar Abbas, Iran; 2 Infectious and Tropical Diseases Research Center, Hormozgan Health Institute, Hormozgan University of Medical Sciences, Bandar Abbas, Iran; 3 Research Center for Health Sciences, Department of Biology and Control of Disease Vectors, School of Health, Institute of Health, Shiraz University of Medical Sciences, Shiraz, Iran; 4 Department of Pharmaceutics, Scholl of Pharmacy, Islamic Azad University, Tehran, Iran; 5 Noncommunicable Diseases Research Center, Fasa University of Medical Sciences, Fasa, Iran; 6 Department of Medical Nanotechnology, School of Advanced Technologies in Medicine, Fasa University of Medical Sciences, Fasa, Iran

**Keywords:** Nanoliposomes, Anopheles stephensi, Culex quinquefasciatus

## Abstract

**Background:**

Mosquito-borne diseases such as malaria and encephalitis are still the cause of several hundred thousand deaths annually. The excessive use of chemical insecticides for transmission control has led to environmental pollution and widespread resistance in mosquitoes. Botanical insecticides' efficacies improvement has thus received considerable attention recently.

**Methods:**

The larvicidal effects of three essential oils from the *Citrus* family and limonene (their major ingredient) were first investigated against malaria and filariasis mosquito vectors. An attempt was then made to improve their efficacies by preparing nanoliposomes containing each of them.

**Results:**

The larvicidal effect of nanoformulated forms was more effective than non-formulated states. Nanoliposomes containing *Citrus aurantium* essential oil with a particle size of 52 ± 4 nm showed the best larvicidal activity (LC_50_ and LC_90_ values) against *Anopheles stephensi* (6.63 and 12.29 µg/mL) and *Culex quinquefasciatus* (4.9 and 16.4 µg/mL).

**Conclusion:**

Due to the green constituents and high efficacy of nanoliposomes containing *C. aurantium* essential oil, it could be considered for further investigation against other mosquitoes’ populations and field trials.

## Background

Mosquitoes are members of the order *Diptera*, class *Insecta*, and phylum *Arthropoda* that live in subtropical or tropical regions [1]. They transmit many diseases such as malaria, dengue fever, yellow fever, encephalitis, and filariasis to humans [2]. For example, it has been estimated that about 229 million new malaria infections and about 0.4 million deaths occurred only in 2019; 67% of death was related to children < 5 years old [3, 4]. *Anopheles stephensi* Liston. is the primary malaria vector in the Indian subcontinent, Arabian Peninsula, Iran, and Afghanistan [5, 6]. On the other hand, *Culex quinquefasciatus* Say, the southern house mosquito, is another geographically widespread and medically important mosquito vector. It transmits viruses such as West Nile and St. Louis encephalitis and the filarial worm, *Wuchereria bancrofti* [7, 8].

Treating such diseases is challenging while preventing their transmission is an accessible and effective way to reduce disease burden and economic, emotional, and health consequences. For example, insecticide-treated nets (ITNs) effectively control malaria in children and adults living in areas with persistent malaria transmission. Residual spraying (IRS) and space spraying of chemical insecticides are also recommended. Besides, larvicide is recommended in endemic regions and regions [9, 10]. However, excessive use of chemical insecticides has led to environmental pollution; toxin residues in agricultural materials or drinking water are health challenges worldwide nowadays [11, 12]. Moreover, in addition to direct adverse effects on human health, they also affect non-target beneficial insects such as bee venom and other pollinating insects [13, 14].

Moreover, repetitive and non-compliant chemical insecticides have also increased mosquitoes' resistance [15, 16]. Herbal insecticides, especially essential oils (EOs), are potential alternatives to chemical ones because they are environmentally friendly, biodegradable, and generally have no toxic effects on non-target organisms [17, 18]. Some scientific studies have also recently been suggested them as alternative agents against mosquito larvae [19, 20]. However, their usage in practical conditions is questioned due to their instability and lower efficacies than chemical larvicide.

The preparation of EO-based nanoformulations is considered a promising approach to prevent oxidative destruction, improve dispersion in water, and enhance the biological efficacies of EOs [21–23]. In the pharmaceutical industry, one commonly used carrier is nanoliposomes; they are vesicles whose structure resembles a cell membrane [24]. Furthermore, due to their hydrophobic nature, they have a higher loading capacity of EOs than chitosan nanoparticles [25, 26]; they are thus proper candidates in developing green larvicide.

This study first investigated the larvicidal effect of the three EOs, including *Citrus aurantium*, *Citrus limon*, *Citrus sinensis*, and limonene (as their identified major ingredients), against *A. stephensi* and *C. quinquefasciatus.* An attempt was then made on enhancing their efficacy by preparation of nanoliposome containing each of them.

## Methods

### Reagents

Limonene (97%), wool fat cholesterol (97%), egg yolk lecithin (> 90%), tween 20 (99.9%), and absolute ethanol (99.9%) were bought from Merck Chemicals (Germany). In addition, *C. aurantium* EO (99.9%) was bought from Tabib Daru pharmaceutical Co. (Iran). *C. limon* EO (99.9%) was purchased from Barij Essence Co. (Iran). *C. sinensis* EO (99.9%) supplied by Green Plants of Life Co.

### Preparation of nanoliposomes

**Fig. 1:**
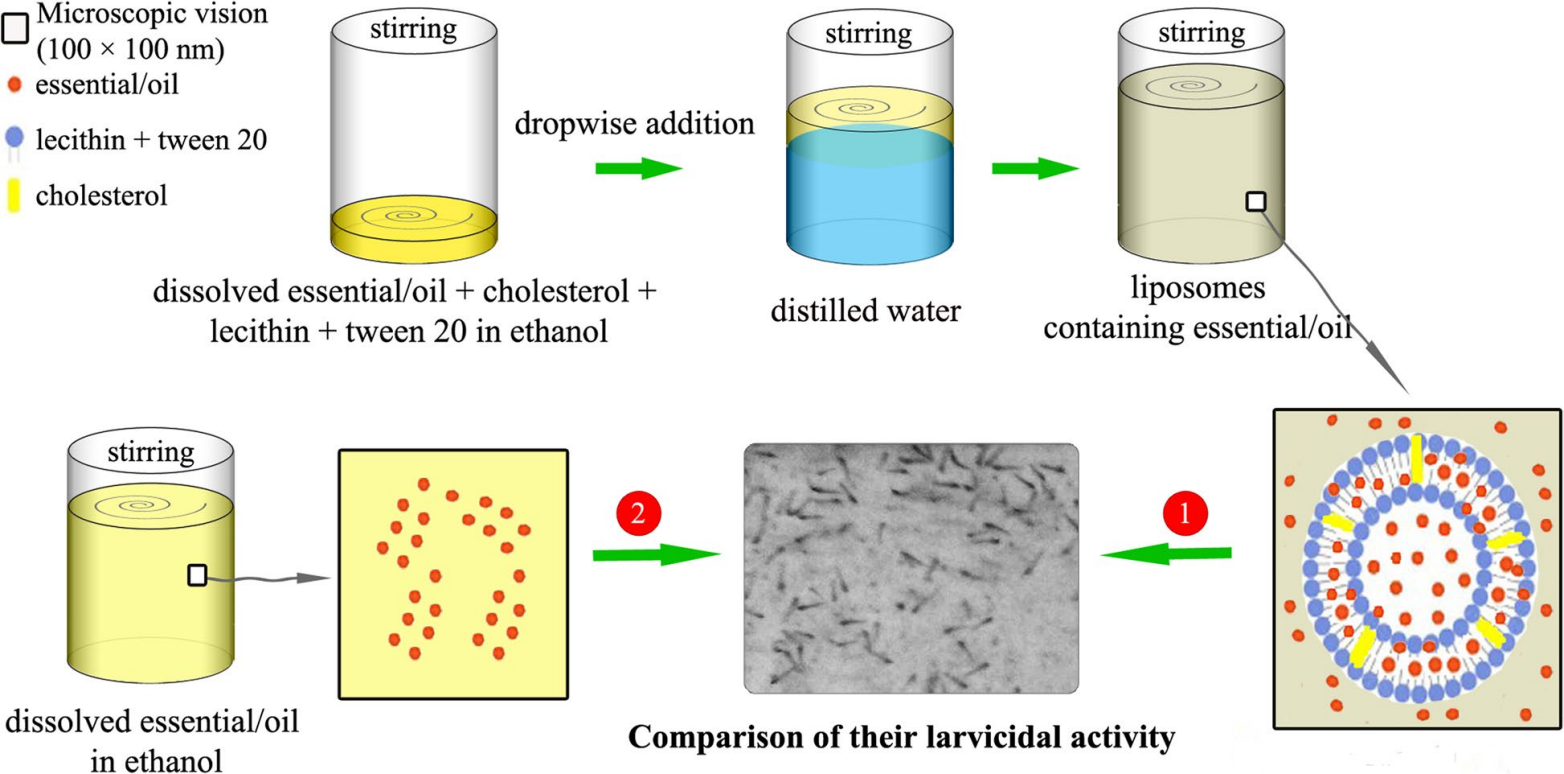
Steps of preparation of nanoliposomes containing limonene, C. aurantium EO, C. limon EO, C. sinensis, and comparison of their larvicidal activities with their non-formulated states (drawn by the corresponding author)

Nanoliposomes containing limonene and each EO were prepared using the ethanol injection approach with some modifications [27]. The lipid phase was first prepared by dissolving lecithin (2.5% w/v), cholesterol (0.5% w/v), tween 20 (1.0% w/v), and limonene, *C. aurantium* EO, *C. limon* EO, *C. sinensis* EO (2% w/v), separately in absolute ethanol. Then, one mL of each prepared solution was added dropwise to 4 mL of distilled water, and the mixtures were stirred for 40 min (2000 rpm, room temperature). The prepared samples were abbreviated as LimLipo, CALipo, CLLipo, and CSLipo, and were used for size analyses, chemical analysis, and larvicidal assays (Fig. 1). Moreover, free liposomes were also prepared similarly, without EOs or limonene.

### Size analyses

The particle sizes of LimLipo, CALipo, CLLipo, and CSLipo were investigated using a dynamic light scattering (DLS) instrument (9900 series, K-One nano Ltd, Korea). The samples' particle size distribution (SPAN) was also calculated as d90-d10/d50, where d is diameter and 10, 50, and 90 are percentages of particles with lower sizes than these values. SPAN values less than 1 confirm narrow particle size distribution [28].

### Confirmation of limonene and EOs in nanoliposomes

Loading of limonene and the EOs in nanoliposomes was investigated using ATR-FTIR analysis (Model Tensor II, Bruker, USA). Free liposomes, LimLipo, CALipo, CLLipo, and CSLipo, were centrifuged for 60 min at 4 ºC (12,000 g). The resulting pellets were placed at room temperature to reduce their moisture for one day. They were then subjected to the instrument, and the spectra were recorded in the range of 400–4000 cm^−1^.

### Larvicidal bioassays

Late third or early fourth instar larvae of *A. stephensi* and *C. quinquefasciatus* (Bandar Abbas strain) were reared in the insectary of Hormozgan University Medical Sciences (Iran). The colonies were maintained at 27 ± 2 ºC with 12:12 light and dark photoperiod at 65% ± 5% relative humidity. Besides, they were not exposed to any insecticides and were susceptible to all larvicides.

Limonene, *C. aurantium* EO, *C. limon* EO, and *C. sinensis* EO were dissolved in ethanol at a concentration equal to as-prepared nanoliposomes (2.5 w/v). Their larvicidal effects as non-formulated samples and LimLipo, CALipo, CLLipo, and CSLipo as nanoformulated samples were investigated according to the WHO guideline [29]. Briefly, beakers containing 200 mL dechlorinated water and 25 *A. stephensi* and *C. quinquefasciatus* larvae were first ready. By adding ≤ 0.8 mL of each sample; concentrations were fixed at 100, 50, 25, 12.50, 6.25, and 3.12 µg/mL. Larval mortality was recorded after 24 h exposure; no food was given to the larvae during these tests. Larvicidal effects of free liposomes were also investigated by adding equal amounts compared with samples. Besides, control groups were exposed to only 0.8 mL ethanol.

### Statistical analyses

All experiments were carried out in triplicate, and final values were reported as mean ± standard deviation. Lethal concentration values (LC_50_ and LC_90_) with 95% fiducial limits and probit equations were calculated using probit analysis, as described by Finney [30]. One-Way-ANOVA or independent sample t-test with a confidence interval of 95% was used (SPSS v.21 software, USA) to compare the samples' larvicidal activity.

## Results

### Size analyses of the prepared nanoliposomes

**Fig. 2:**
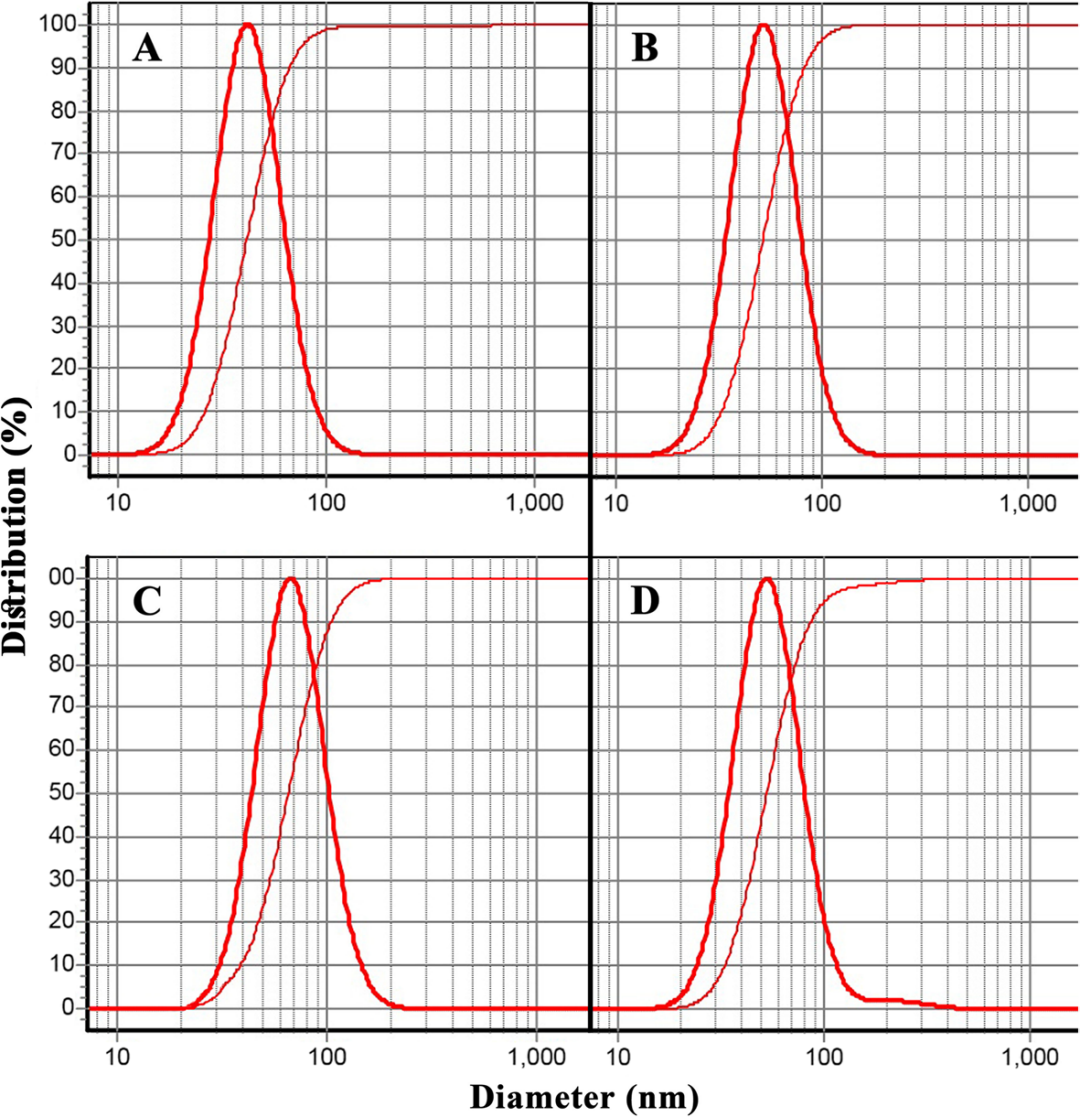
DLS analyses of the nanoliposome containing limonene 42 ± 5 nm **A**, and EOs of C. aurantium 52 ± 4 nm **B**, C. limon 67 ± 5 nm **C**, and C. sinensis 53 ± 7 nm **D**

DLS analyses of the prepared nanoliposomes containing limonene, *C. aurantium* EO, *C. limon* EO, and *C. sinensis* EO are depicted in Fig. 2; their particle size was in the range of 42–67 nm. Their uniformity was confirmed as their particles size distributions (SAPN) were obtained as 0.97, 0.94, 0.98, and 0.99. Besides, the presence of a single sharp peak is also a sign of uniformity of nanoparticles.

### Loading of limonene and EOs into nanoliposomes

**Fig. 3:**
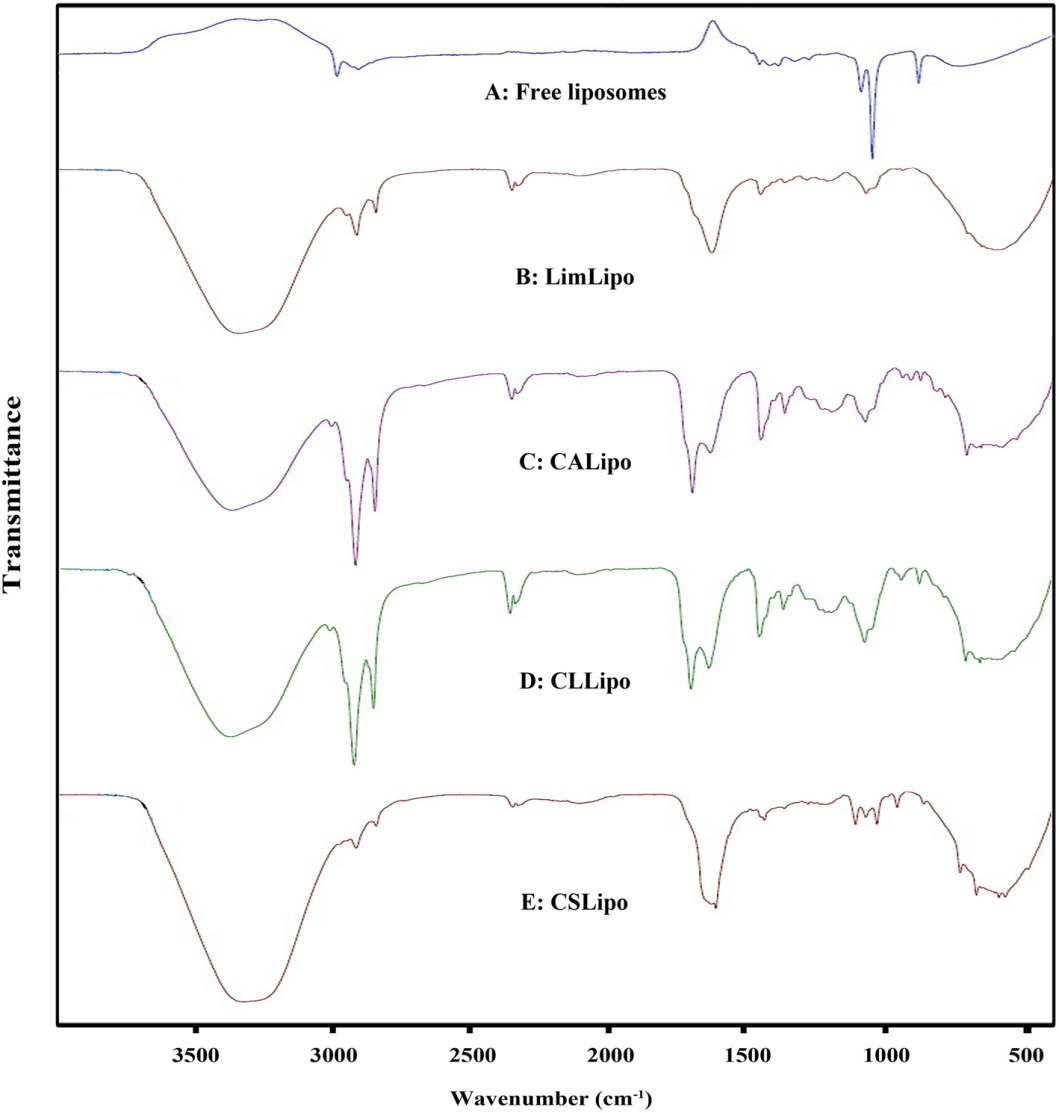
ATR spectra of free liposomes **A**, nanoliposomes containing limonene **B**, nanoliposomes containing C. aurantium EO **C**, nanoliposomes containing C. limon EO **D**, and nanoliposomes containing C. sinensis EO **E**

ATR-FTIR spectra of free liposomes (Fig. 3A) display the bands at 2981 and 2904 cm^−1^; they attribute to C–C-H stretching (alkane groups). The band at 1453 cm^−1^ is related to CH_2_ bending, and the characteristic absorption at around 1385 cm^−1^ shows CH_3_ bending. The band at 1274 cm^−1^ is assigned to PO_2_ groups in lecithin. The band at 877 cm^−1^ is attributed to N(CH_3_)_3,_ the band at 1085 cm^−1^ is attributed to P = O, the strong band at 1044 cm^−1^ is related to C-O stretching.

In limonene-loaded nanoliposomes (Fig. 3B; LimLipo), expanding the peak in 3345 cm^−1^ is due to increased hydrogen bands between tween 20, OH of cholesterol, and carbonyl groups (C = O) of fatty acid esters in lecithin structure. The bands at 2955, 2918, and 2850 cm^−1^ show –CH stretching in alkane groups. The peak at 1637 cm^−1^ relates to binding between lecithin and cholesterol, the band at 1225 cm^−1^ is assigned to PO_2_ groups in lecithin, and the band at 961 cm^−1^ is attributed to N(CH_3_)_3_. The most characteristic peak at about 886 cm^−1^ is attributed to the terminal methylene group out of plane bending in limonene. All the bands attributed to the functional groups of limonene and liposomes, including (methyl, hydroxyl, ester) were also presented in the limonene-loaded nanoliposome spectrum.

In the ATR-FTIR spectra of CALipo (Fig. 3C), the broadband at about 3369 cm^−1^ is related to OH stretching vibrations that confirmed increasing hydrogen bands (tween, OH cholesterol, and carbonyl groups of fatty acid esters in lecithin structure). The bands at 2922 and 2852 cm^−1^ correspond to the –CH stretching (alkyl chains in lecithin, cholesterol, and *C. aurantium* EO). The peak at 1708 cm^−1^ corresponds to the stretching vibration of carbonyl C = O, the absorption band at 1644 cm^−1^ assigns to binding between lecithin and cholesterol. The band at 1208 cm^−1^ is attributed to PO_2_ groups in lecithin, and the band at 961 cm^−1^ is assigned to N(CH_3_)_3_. The main characteristic peak in *C. aurantium* EO and limonene is related to the terminal methylene group out of plane bending at 887 cm^−1^.

The ATR-FTIR spectra of CLLipo (Fig. 3D) showed the broadband at about 3367 cm^−1^ which could be attributed to OH stretching vibrations; this broadening of infrared spectra is related to forming hydrogen bonds in tween, OH of cholesterol, and carbonyl groups of fatty acid esters in lecithin structure. The bands at 2921 and 2851 cm^−1^ show –CH stretching vibration related to alkane groups. The strong peak at 1708 cm^−1^ corresponds to the stretching vibration of carbonyl C = O, and the peak at 1645 cm^−1^ could be related to the binding between lecithin and cholesterol. The peak at 1206 cm^−1^ corresponds to PO_2_ groups in lecithin, the band at 962 cm^−1^ is related to N(CH_3_)_3_. The peak at about 886 cm^−1^ is attributed to the terminal methylene group out of plane bending in limonene as the main compound of *C. limon* EO.

The ATR-FTIR spectra of CSLipo (Fig. 3E) displayed the broadband at about 3331 cm^−1^. It relates to OH stretching vibrations in hydrogen-bonded (tween, OH of cholesterol, and carbonyl groups of fatty acid esters in lecithin structure). The band at 1222 cm^−1^ corresponds to PO_2_ groups in lecithin, and the absorption bands at 2924 and 2852 cm^−1^ relate to alkane groups stretching. The strong peak at 1626 cm^−1^ could be related to the binding between lecithin and cholesterol. The band at 972 cm^−1^ corresponds to N(CH_3_)_3_. The peak at about 877 cm^−1^ is assigned to the terminal methylene group out of plane bending in limonene as the main compound of *C. sinensis* EO. Eventually, the peak at about 886 cm^−1^ was evidence of limonene presence. The ATR-FTIR analyses of nanoliposomes containing EOs and limonene have made us notice a similitude of absorption bands and merely small differences between their intensities. This result also confirmed only physical interactions between EOs or limonene and free liposomes.

### Larvicidal effect of samples

**Fig. 4:**
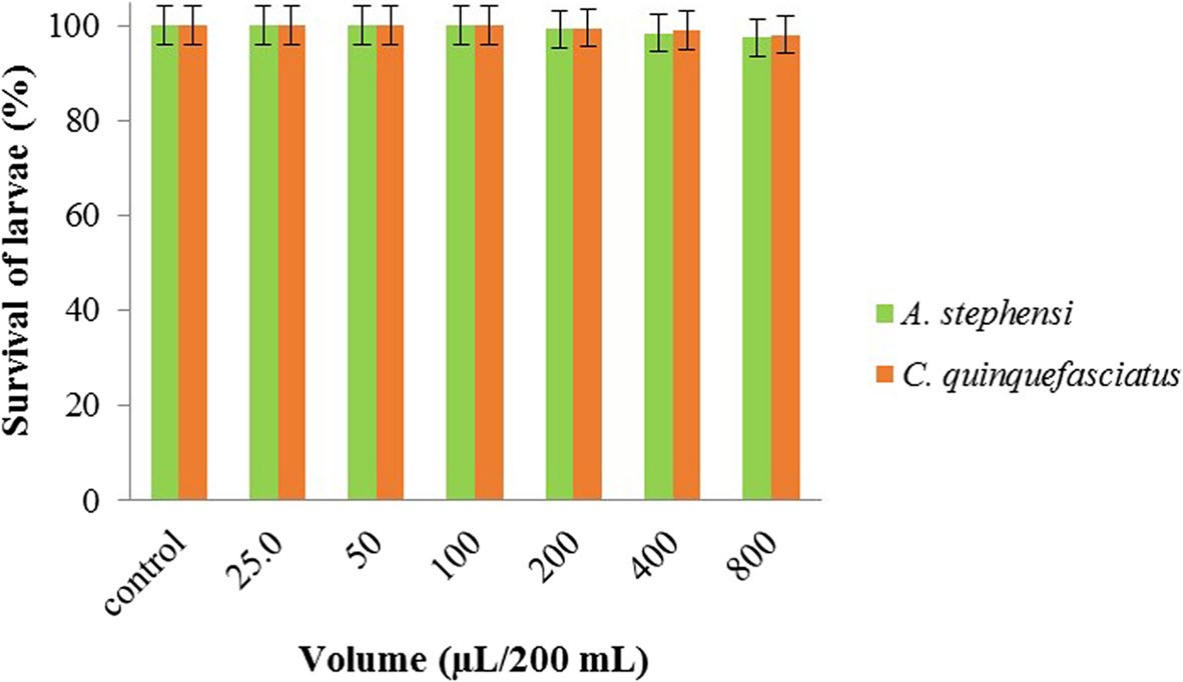
Survival of A. stephensi and C. quinquefasciatus larvae after 24 h exposure with different amounts of free nanoliposomes (without EOs or limonene)

As shown in Fig. 4, free liposomes did not affect the larvae. Viabilities of both *A. stephensi* and *C. quinquefasciatus* were 97–100% after treatment with different amounts of free liposomes, equal to the values of the samples containing EOs or limonene were treated.

Probit regression lines parameters of *A. stephensi* larvae exposed to different concentrations of samples are listed in Table 1. Among the non-formulated samples (limonene, *C. aurantium* EO, *C. limon* EO, and *C. sinensis* EO), LC_50_ and LC_90_ values of *C. aurantium* EO were significantly higher than others (one-way ANOVA, *P* < 0.05). The best-observed LC_50_ values (6.63 µg/mL) and LC_90_ value were related to CALipo (12.29 µg/mL).

**Table 1:** Probit regression line parameters of *A. stephensi* exposed to the samples

Specimens	A	B ± SE	LC_50_	LC_90_	χ^2^ (df)	*P*-value	Probit Equation Y = A + BX
			**(LCL-UCL)**				
**limonene**	-2.79	2.14 ± 0.441	20.12 (9–40)	80.05 (40–106)	14.91 (3)	< 0.05	Y = -2.7853 + 2.1367 X
^**a**^**LimLipo**	-5.46	4.82 ± 0.470	13.6 (12–15)	25.08 (22–30)	3.32 (2)	> 0.05	Y = -5.4638 + 4.8201 X
***C. aurantium* EO**	-3.52	1.96 ± 0.214	62.49 (52–80)	281 (190–506)	4.76 (3)	> 0.05	Y = -3.5249 + 1.9628 X
^**b**^**CALipo**	-3.93	4.79 ± 0.468	6.63 (6–7)	12.29 (11–15)	1.78 (2)	> 0.05	Y = -3.9348 + 4.7884 X
***C. limon* EO**	-3.82	3.34 ± 0.322	13.87 (12–16)	33.53 (28–42)	4.74 (2)	> 0.05	Y = -3.8173 + 3.3427 X
^**c**^**CLLipo**	-3.08	3.7 ± 0.353	6.8 (6–8)	15.12 (13–19)	2.26 (2)	> 0.05	Y = -3.0788 + 3.6969 X
***C. sinensis* EO**	-2.95	2.7 ± 0.811	12.41 (0.5–36)	37.03 (18- 59)	29.65 (3)	< 0.05	Y = -2.9517 + 2.6987 X
^**d**^**CSLipo**	-6.41	6.53 ± 0.705	9.6 (9–10)	15.08 (13–18)	0.43 (2)	> 0.05	Y = -6.4103 + 6.5271 X

*A* y-intercept, *B* the slope of the line, *SE* Standard error, *LC*_*50*_ Lethal concentration causing 50% mortality (µg/mL), *LC*_*90*_ Lethal concentration causing 90% mortality, *LCL* Lower Confidence Limit (95%), *UCL* Upper Confidence Limit (95%), *χ*^*2*^ heterogeneity about the regression line, *df* degree of freedom, *p* value represent heterogeneity in the population of tested

^a^nanoliposomes containing limonene

^b^nanoliposomes containing *C. aurantium* EO

^c^nanoliposomes containing *C. limon* EO

^d^nanoliposomes containing *C. sinensis* EO

However, LC_50_ values of nanoformulations were lower than their non-formulated state; this difference was only significant between *C. limon* EO and CLLipo as well as *C. aurantium* EO and CALipo (Independent sample t-test, *P* < 0.05). Besides, the LC_90_ values of all nanoformulations were significantly more deleterious to larvae than their non-formulated forms (Independent sample t-test, *P* < 0.05).

Probit regression lines parameters of *C. quinquefasciatus* exposed to different concentrations of samples are listed in Table 2. Among the non-formulated samples, the LC_50_ value of limonene was higher than others; however, this difference was only significant compared to *C. sinensis* EO (one-way ANOVA, *P* < 0.05). Besides, LC_90_ values of all non-formulated samples were not significantly different together (one-way ANOVA, *P* > 0.05). In all samples, the best-observed LC_50_ (4.9 µg/mL) and LC_90_ (16.4 µg/mL) values were related to CALipo.

**Table 2:** Probit regression line parameters of *C. quinquefasciatus* exposed to the samples

Specimens	A	B ± SE	LC_50_	LC_90_	χ^2^ (df)	*P*-value	Probit Equation Y = A + BX
			**(LCL-UCL)**				
**limonene**	-5.52	4.55 ± 0.428	16.36 (15–18)	31.29 (27–38)	9.93 (2)	> 0.05	Y = -5.523 + 4.5503 X
^**a**^**LimLipo**	-3.48	4.31 ± 0.418	6.41 (6–7)	12.71 (11–15)	24.68 (2)	> 0.05	Y = -3.4769 + 4.309 X
***C. aurantium* EO**	-6.74	5.61 ± 0.570	15.9 (15–17)	26.9 (24–32)	2.01 (2)	> 0.05	Y = -6.7419 + 5.6119 X
^**b**^**CALipo**	-1.69	2.45 ± 0.497	4.9 (2–8)	16.4 (10–18)	11.15 (3)	< 0.05	Y = -1.6887 + 2.4455 X
***C. limon* EO**	-5.6	4.78 ± 0.459	14.87 (13–16)	27.6 (24–32)	20.32 (2)	> 0.05	Y = -5.6018 + 4.7774 X
^**c**^**CLLipo**	-3.11	2.97 ± 0.273	11.15 (10–13)	30.15 (25–38)	3.66 (3)	< 0.05	Y = -3.1083 + 2.9675 X
***C. sinensis* EO**	-4.94	4.44 ± 1.6	12.99 (12–14)	25.24 (22–30)	27.57 (2)	< 0.05	Y = -4.9439 + 4.4401 X
^**d**^**CSLipo**	2.031	1.99 ± 0.191	10.48 (9–12)	46.15 (35–67)	4.78 (3)	> 0.05	Y = -2.0309 + 1.9905 X

*A* y-intercept, *B* the slope of the line, *SE* Standard error, *LC*_*50*_ Lethal concentration causing 50% mortality (µg/mL), *LC*_*90*_ Lethal concentration causing 90% mortality, *LCL* Lower Confidence Limit (95%), *UCL* Upper Confidence Limit (95%), *χ2* heterogeneity about the regression line, *df* degree of freedom, *p* value represent heterogeneity in the population of tested

^a^nanoliposomes containing limonene

^b^nanoliposomes containing *C. aurantium* EO

^c^nanoliposomes containing *C. limon* EO

^d^nanoliposomes containing *C. sinensis* EO

Furthermore, the LC_50_ values of LimLipo, CALipo, and CLLipo were significantly more effective than their non-formulated states (Independent sample t-test, *P* < 0.05); no significant difference was seen between *C. sinensis* EO and CSLipo (Independent sample t-test, *P* > 0.05). LC_90_ values of LimLipo and CALipo were significantly more efficient than their non-formulated states (Independent sample t-test, *P* < 0.05).

## Discussions

As detailed in our previous report, limonene was identified as the major compound in the used EOs; it composed 31.4, 61.8, and 61.8% of identified compounds in *C. aurantium*, *C. limon*, and *C. sinensis* EOs [31]. Sabinene (15.6%), ɣ-terpinene (6.0%), linalool (5.6%), and nerolidol (5.1%) were the other four major compounds of *C. aurantium* EO. Alpha-pinene, sabinene, *cis*-limonene oxide, and *trans*-limonene oxide with a portion of 3.5, 17.0, 2.3, and 3.1% were the other four *C. limon* EO compounds. In *C. sinensis* EO, *trans*-p-2,8-Menthadien-1-ol, cis-limonene oxide, *trans*-limonene oxide, and *trans*-carveol were identified as the other four major compounds (5.0, 2.6, 2.3, and 2.9%). Limonene is a colorless terpene with a pleasant lemon-like odor [32, 33].

The literature reported a nanoliposome containing limonene with a particle size of 100 nm; however, it was used as an antibacterial agent [34]. In another research, liposomes containing *C. limon* EO with a particle size of 114 nm was also reported [35]. Interestingly, we could not find any report on the preparation of liposomes containing *C. sinensis* and *C. aurantium* EOs.

The larvicidal effects of *C. aurantium* EO were previously reported; the LC_50_ value against *A. stephensi* was 31.20 ppm, but details on *C. quinquefasciatus* were not available [36, 37]. However, in the current study, LC_50_ of *C. aurantium* EO was observed at 62.49 μg/mL; the difference was related to the used strain of *A. stephensi* (Bandar Abbas vs. Beech). Besides, the LC_50_ value of *C. sinensis* EO against larvae of *C. quinquefasciatus* in two separate reports was reported at 304 and 452 ppm; however, used strains were not mentioned [38, 39]. Furthermore, no report was found on the larvicidal effect of limonene and *C. limon* EO, LimLipo, CLLipo, CALipo, and CSLipo against *A. stephensi* and *C. quinquefasciatus*. The present research is thus a new venture into this specific field. Interestingly, the best-obtained result in the current study was related to nanoliposomes containing *C. aurantium* EO with LC_50_ values of 6.63 and 4.9 μg/mL against larvae *A. stephensi* and *C. quinquefasciatus*. These values were more potent than available reports as EO-based nano larvicides. For instance, nanocrystal emulsion of *Ficus glomerata* with LC_50_ value of 17 μg/mL against *A. stephensi* [40]. Moreover, LC_50_ values of *Azadirachta indica* and *Lippia alba* EOs nanoemulsions were reported at 11.75 and 38.22 μg/mL against *C. quinquefasciatus* [41, 42].

The current data also emphasize the superior activity of nanoformulations over their non-formulated states; it is consonant with the literature. When a solute such as EO is dissolved in the appropriate solvent, droplet size depends on the solution's preparation conditions (temperature and time) is around 0.3 – 10 nm [43, 44]. On the other hand, many droplets are incorporated into a nanocarrier (e.g., nanoliposome) during the formulating process; due to the presence of packages containing EOs, the larvicidal efficacies are improved [45–47]. Furthermore, nanostructures' high surface energy leads to a more efficient interaction between nanoparticles and the target organism [48]. Besides, nanosize carriers improve EO's ability to pass through tiny pores in larval bodies [49]. The higher physical stability of nanoformulated EO could also improve larvicidal effects [50].

Scanning the literature, the effect of nanoformulations compared to non-formulated EOs could be summarized as follows. First, achieved a 100% larvicidal effect at lower exposure time, *e.g.,* the study conducted on *C. quinquefasciatus* a perfect larvicidal effect was achieved at low exposure time, *Eucalyptus globulus* EO (24 h), and its nanoemulsion (4 h) [51]. Second, an improvement of LC_50_ value of EOs was acquired after preparing their nanoformulated form; for instance, the LC_50_ value of *C. sinensis* EO against *Culex pipiens* was reported at 86.3 μg/mL, and its nanoemulsion dosage form had its LC_50_ value at 27.4 μg/mL [52]. However, few reports on the preparation of slow and long-release nanoformulations and the long-lasting effects of green nanolarvicides were also reported [45, 53, 54].

## Conclusions

The results show that the nanoliposomes containing limonene and limonene-rich EOs were more toxic than non-nanoliposomes (max 20 folds) against malaria and filariasis mosquito larvae. Interestingly, nanoliposomes containing *C. aurantium* EO with LC_50_ values of 6.63 and 4.9 μg/mL against larvae *A. stephensi* and *C. quinquefasciatus* could be considered an alternative to synthetic insecticides for the prevention of transmission of such dreadful diseases. However, by investigating its efficacies against other medically important mosquito species, its effectiveness could be better evaluated.

## Data Availability

All data generated or analyzed during this study are included in this published article.

## Abbreviations

ATR-FTIR: Attenuated Total Reflection-Fourier Transform InfraRed
EO: Essential Oil
LimLipo: Nanoliposomes containing limonene
CALipo: Nanoliposomes containing Citrus aurantium essential oil
CLLipo: Nanoliposomes containing Citrus limon essential oil
CSLipo: Nanoliposomes containing Citrus sinensis essential oil
